# CURRENT PRACTICES OF DRIVING REHABILITATION SPECIALISTS: IS FITNESS TO DRIVE EVALUATIONS USING THE BEST EVIDENCE?

**DOI:** 10.1093/geroni/igae098.1310

**Published:** 2024-12-31

**Authors:** Anne Dickerson

**Affiliations:** East Carolina University, Greenville, North Carolina, United States

## Abstract

It is well established that social participation is essential for maintaining quality of life and in the US, driving is the primary mode of community mobility for older adults. Research has established the importance of fitness-to-drive (FTD) evaluations for those older adults with medical conditions. These medically-at-risk drivers, at any age, are generally evaluated by specialized driver rehabilitation specialists (DRS) to determine FTD. In the last 10 years, much research has established which assessments best determine FTD with cognitive assessments being the primary determinant. The aim of this research study was to compare assessment tools with the best evidence with the actual tools being currently used by DRSs. A comprehensive review of tools was completed by 321 practicing DRSs. Results show that cognitive tools such as Trails A, Trails B, and clock drawing are being used by 91%, 92%, and 74% respectfully. However, other tools with strong evidence are being used much less (MOCA-46%, UFOV-30%). Additionally, assessment with limited or no evidence (e.g., Traffic signs-66%, brake reaction-71%) or with vehicle technology changes, are not essential to FTD (e.g., muscle strength-91%, ROM-95%) are used extensively. Highlights from the research will include how the on-road assessment is used as a determining factor for FTD with statements. For example: If a driver with moderate dementia passes the on road, he/she should be able to continue driving. While 71% indicted this was probably/definitely false, 29% felt it was probably/definitely true. Implications of these results will be discussed.

